# Surgical outcomes of acetabular fracture of elderly patients with superomedial dome impaction

**DOI:** 10.1038/s41598-023-46652-5

**Published:** 2023-11-04

**Authors:** Eic Ju Lim, Hyun-Chul Shon, Jae-Young Yang, Joosuk Ahn, Jung Jae Kim, Ji Wan Kim

**Affiliations:** 1 Department of Orthopedic Surgery, Chungbuk National University Hospital, Chungbuk National University College of Medicine, Cheongju, Republic of Korea; 2 Department of Orthopedic Surgery, Korean Armed Forces Capital Hospital, Gyeonggi-do, Republic of Korea; 3 Department of Orthopedic Surgery, Asan Medical Center, University of Ulsan College of Medicine, 88, Olympic-ro, 43-gil, Songpa-gu, Seoul, Republic of Korea

**Keywords:** Musculoskeletal system, Medical research, Outcomes research

## Abstract

This study aimed to investigate the outcomes of elderly acetabular fractures according to the reduction of impacted dome fragments. A retrospective cohort study was performed in two institutions. Fifty-four patients aged ≥ 60 years with acetabular fractures were enrolled. Data for dome impaction and postoperative reduction was collected. Patients were divided into the good reduction group (displacement ≤ 3 mm) and poor reduction group (displacement > 3 mm). Postoperative osteoarthritis (OA), Harris hip score (HHS), total hip arthroplasty conversion, good/poor outcomes were compared between the two groups. The good reduction group (N = 45) demonstrated a lower proportion of radiographic OA (18 vs. 77%, *P* = 0.001), higher HHS (82.1 vs. 68.6, *P* = 0.022), and higher proportion of good outcomes than the poor reduction group (N = 9) (89 vs. 22%, *P* < 0.001). In a subgroup analysis of the patients with dome impaction, the good reduction group had a higher proportion of good outcomes (80 vs. 20%, *P* = 0.031). On comparing within the good reduction group, dome impaction did not influence clinical outcomes. Elderly acetabular fractures demonstrated favorable outcomes when adequate reduction was achieved even with dome impaction. Well-reduced dome impaction could achieve satisfactory outcomes in elderly acetabular fractures.

## Introduction

With general population aging, the incidence of acetabular fractures in elderly patients increases^1,2^. Although open reduction and internal fixation (ORIF) is the gold standard for younger populations, no consensus on the ideal treatment for elderly acetabular fractures has been established. Because elderly patients have several vulnerabilities to be considered such as pre-injury health status and poor bone quality, several alternative treatments include conservative treatment, ORIF including percutaneous fixation, total hip arthroplasty (THA) with concomitant column fixation, and planned delayed THA^3–6^.

Considering patient factors and injury factors, treatment for acetabular fractures should be highly individualized^7^. Patient factors include pre-injury ambulatory function; medical state such as whether surgery can be tolerated; and injury factors including injury mechanism, associated injury, and fracture characteristics. Regarding fracture characteristics, previous studies have emphasized negative predictive factors such as concomitant femoral head fracture, articular comminution, and articular impaction involving the weight-bearing dome^8,9^. Rommens et al. described ORIF as a way of reconstructing a stable socket for later safe-cup implantation when negative predictive factors present in elderly patients^10^.

**Figure 1:**
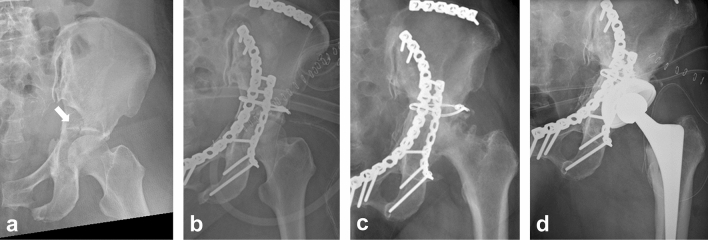
Case of acetabular fracture with dome impaction. (**a**) A 72-year-old woman had an injury associated with a column fracture with dome impaction (white arrow). (**b**) Surgery was performed without reduction and fixation of the impacted dome fragment. (**c**) Five years postoperatively, the patient presented with post-traumatic osteoarthritis and severe pain. (**d**) Total hip arthroplasty was performed.

Among those negative predictive factors, superomedial dome impaction (dome impaction), described as ‘gull sign’ by Anglen et al., was initially reported as the inability to obtain reduction and stable fixation^11^ (Fig. 1). They have reported that early loss of reduction occurred in 7 of 10 patients with dome impaction despite reduction of the dome fragment, and dome impaction was 100% predictive of failure of reduction and/or fixation. Subsequently, reduction techniques for dome impaction have been reported with relatively successful outcomes^12^. Laflamme et al. have demonstrated direct reduction through an intrapelvic approach, reporting 33% (3 out of 9 patients) of overall conversion rate to THA^13^.

However, studies on the comparative outcomes of reduction of dome impaction in elderly patients are few. Therefore, we hypothesized that despite dome impaction, adequate reduction would improve clinical outcomes. The present study aimed to investigate the outcomes of elderly acetabular fractures according to reduction of impacted dome fragments.

## Materials and methods

This study was performed in line with the principles of the Declaration of Helsinki. This study was approved by our institutional review board and a waiver of the requirement for written informed consent was granted (Asan Institute for Life Science, approval no.: 2021–3165-0003). Data collection was performed in accordance with relevant guidelines and regulations issued by the committee.

### Patient selection

**Figure 2:**
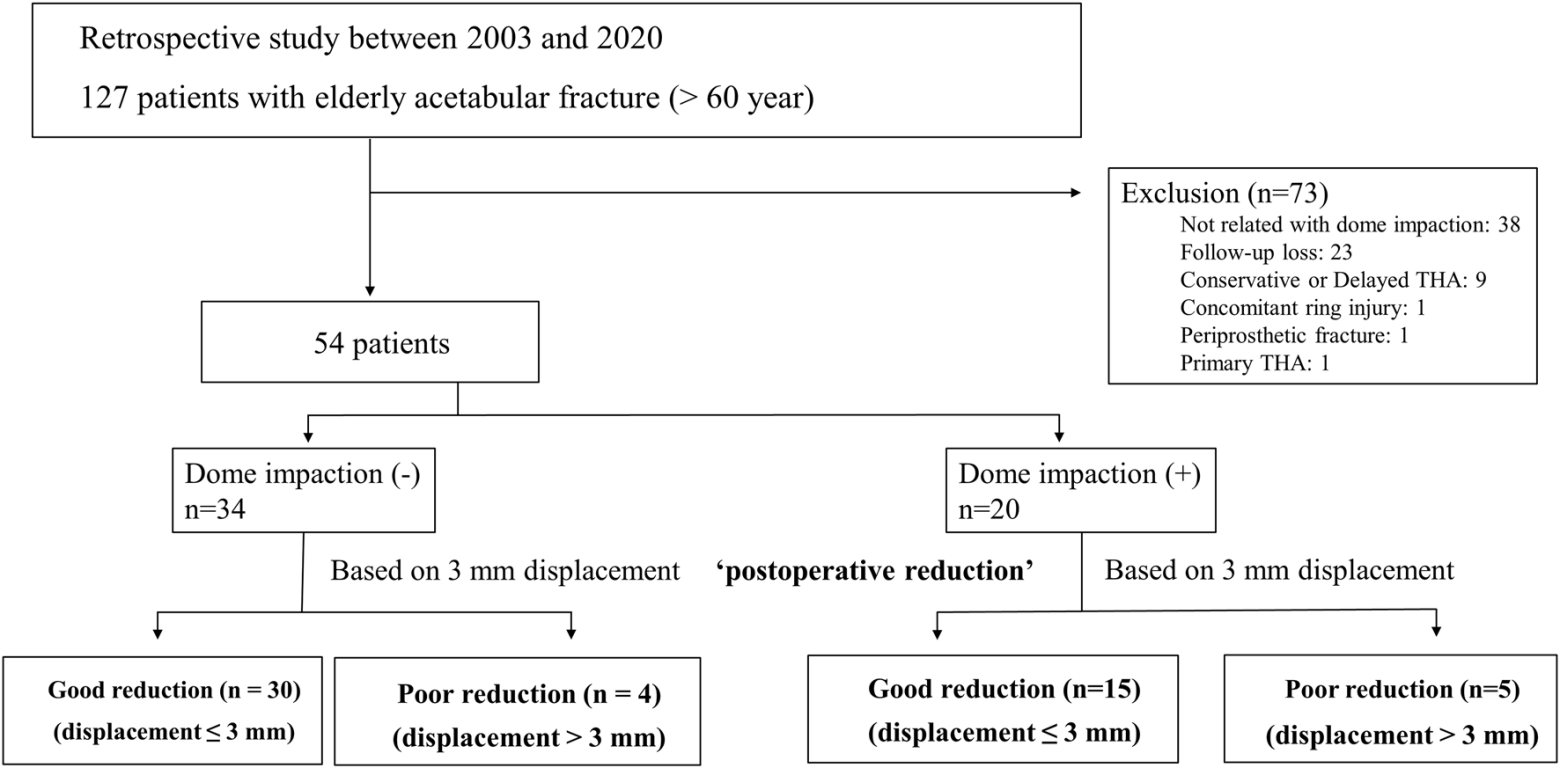
Flowchart of patient enrollment and grouping. THA, total hip arthroplasty.

This retrospective comparative study was conducted in two university teaching hospitals. The inclusion criteria were as follows: patients aged ≥ 60 years, patients with acute acetabular fractures, and patients who had received surgical treatment. Initially, 127 patients were enrolled from the pelvis and acetabular fracture cohort database from 2003 to 2020. Then, we excluded 73 patients with fracture patterns unrelated with dome impaction (anterior wall, posterior wall, posterior column, and posterior column with posterior wall), who were treated conservatively or with acute THA, had concomitant pelvic ring injury, had periprosthetic fractures, and were followed up for < 12 months. Fracture patterns which can contain dome impaction include anterior column, transverse, transverse and posterior wall, T-type, anterior column and posterior hemitransverse, and both column fractures. Finally, 54 patients were enrolled in the present study (Fig. 2).

### Surgical technique for reduction of dome impaction

The surgeries were performed by three operators (H.-C.S., J.J.K, and J.W.K.) with more than 10 years of experience performing pelvic and acetabular fracture surgeries. The patient was placed in a supine position with silicone padding under the knee to flex the hip and knee joints so that the iliopsoas muscle, external iliac vessel, and femoral nerve could relax. Skeletal traction at the distal femur was prepared to facilitate reduction of central dislocation of femoral head and exposure of acetabular joint surface.

**Figure 3:**
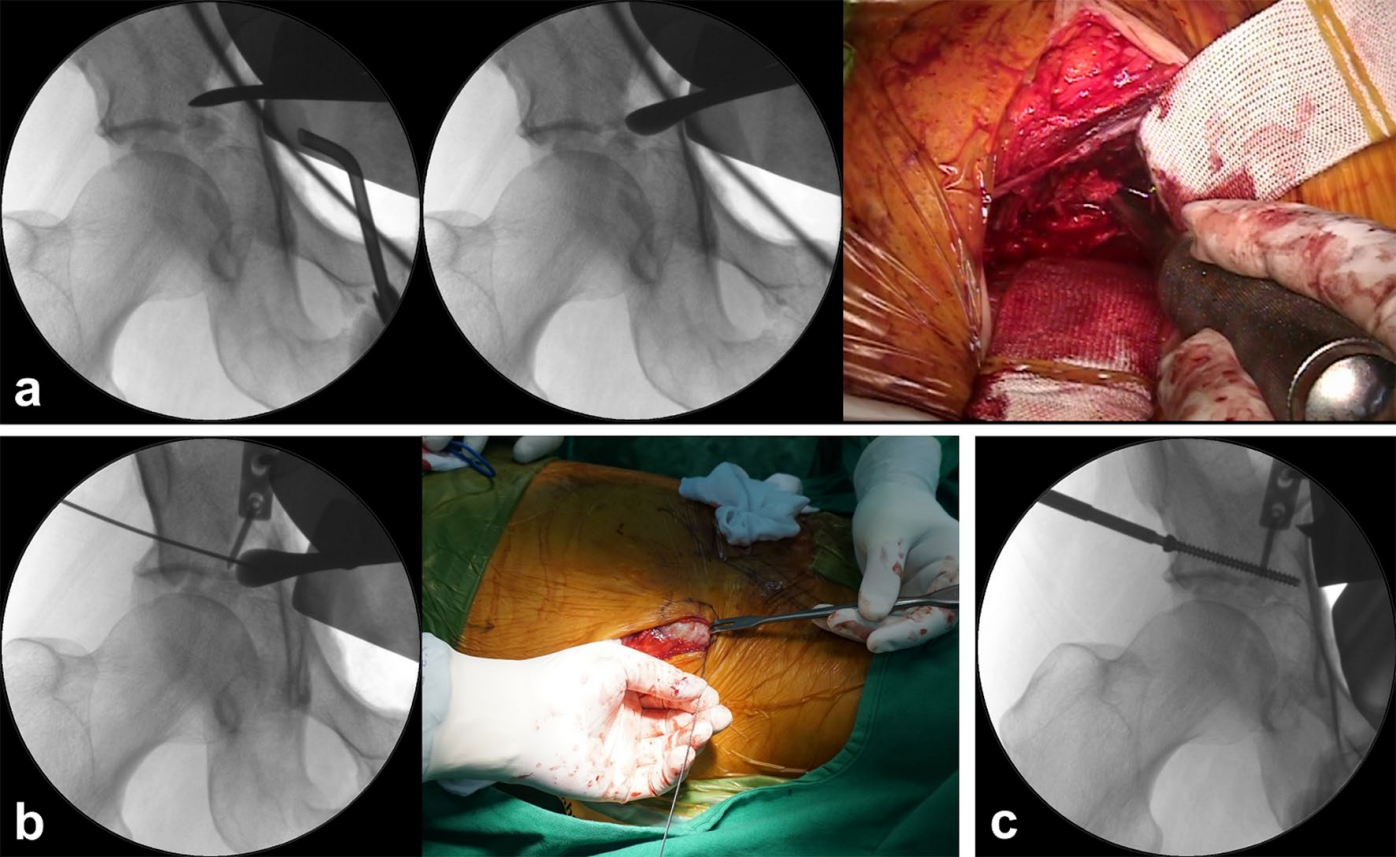
Surgical procedure for reduction of dome impaction. (**a**) The impacted dome fragment was reduced using an elevator. (**b**) The reduced dome fragment was temporarily fixed using a K-wire. (**c**) A 3.5-mm cannulated screw was inserted along the K-wire as a guide pin.

After identifying the obturator nerve through the intrapelvic approach, the fracture site between the anterior column and quadrilateral plate was exposed. Hematoma and soft tissue in the fracture site were removed carefully, and an elevator was inserted through the fracture site to confirm the position of the dome fragment in the C-arm. Then, the impacted dome fragment was reduced using an elevator and temporarily fixed using a K-wire (Fig. 3).

Through a lateral window, the anterior column was reduced and fixed using a five-hole reconstruction plate or a 3.5-mm locking compression plate. The K-wire for a cannulated screw was started 1.5 cm downward and 1.5 cm medially from the anterior superior iliac spine in the lateral window to fix the dome fragment directly or provide support with a rafting screw in tibial plateau surgery. The K-wire was inserted only to cross the dome fragment without penetrating the quadrilateral surface because the K-wire could interfere with the reduction of the quadrilateral surface if it was fully inserted before reduction.

The quadrilateral surface was reduced using a ball and spike or collinear clamp and was then penetrated by the K-wire. A 3.5-mm cannulated screw was inserted along the K-wire which was used as a guide pin for the cannulated screw. The anterior column was stabilized with a long reconstruction plate, and the posterior column was fixed using a posterior column screw if necessary.

After postoperative recovery, active and passive knee and hip range of motion exercises were commenced. Wheelchair ambulation was encouraged as soon as possible if the pain was tolerable. Afterward, weight-bearing was gradually permitted according to the patient’s condition and fracture pattern.

### Data collection and reduction evaluation

Data on demographics, including age; sex; body mass index (BMI); age-adjusted Charlson comorbidity index (CCI)^14^; injury mechanism; the lowest T-score for the lumbar spine, neck, and total femur of the bone mineral density; preoperative osteoarthritis (OA); and follow-up period, were assessed. A low-energy mechanism of injury was defined as falls from heights of ≤ 1 m^15^. Preoperative OA was evaluated in the fractured hip whenever possible using Kellgren and Lawrence (K–L) grade^16^ by two authors (E.J.L. and J.A.). When the evaluation was difficult due to intra-articular fracture, the OA grade of the contralateral hip joint was used. Fracture pattern was evaluated based on the Judet and Letournel classification^16^. Approaches used for the acetabular fracture surgery were evaluated using surgical records. The presence of negative predictive factors, such as posterior wall involvement, dome impaction, femoral head impaction, posterior dislocation, central dislocation, and separation of the quadrilateral plate was evaluated through a preoperative computed tomography (CT) scan.

**Figure 4:**
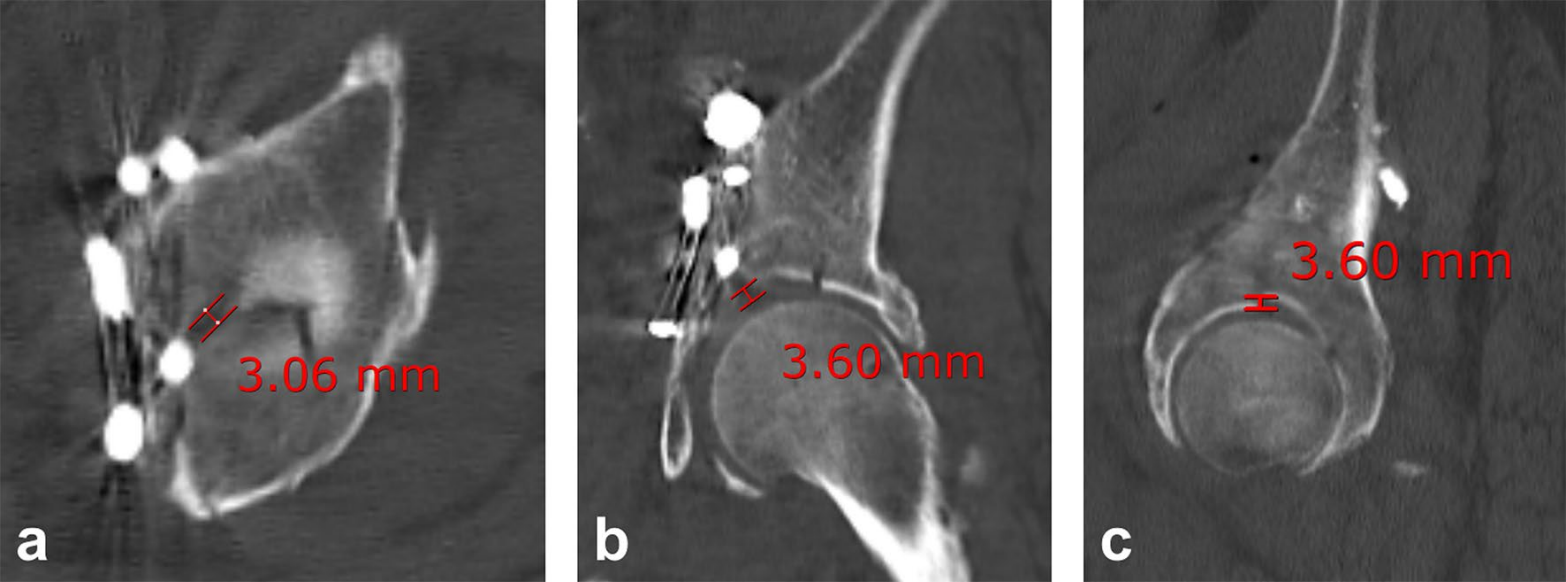
Evaluation of quality of reduction. (**a**) Gap and (**b** and **c**) step displacement in the weight-bearing dome was evaluated based on postoperative computed tomography with (**a**) axial, (**b**) coronal, and (**c**) sagittal planes.

Quality of reduction was evaluated according to the Matta criteria^17^. We evaluated gap and step displacement in the weight-bearing dome based on the pelvis CT scan with axial, coronal, and sagittal planes^18^. Patients with anatomic and imperfect displacement of ≤ 3 mm according to the Matta criteria were classified as the ‘good reduction group’ and those with poor displacement of > 3 mm as the ‘poor reduction group’ (Fig. 4).

### Outcome assessment and comparison

Postoperative radiographs at the last follow up or immediately before THA were used to assess postoperative OA using the K–L grade. Radiographic OA was defined as K–L grades 3 and 4. Functional score was assessed using the Harris hip score (HHS)^19^ at the final outpatient visit or before THA conversion. Patients without functional scores at the outpatient visit were interviewed by telephone. Conversion to THA was assessed using final radiographs and telephone interview.

The outcomes were categorized dichotomously using radiographic grade and functional score. Poor outcomes were defined as one of the following: conversion to THA or the presence of radiographic OA, combined with poor or fair results in the HHS (HHS < 80). The HHS value is based on the 15th percentile of HHS score following acetabular fracture, which represents the THA conversion after acetabular fracture^21–23^.

Therefore, a good outcome was defined as requiring no additional surgical treatment, good or excellent results in the HHS (HHS ≥ 80), or no evidence of radiographic OA. Deceased patients were evaluated by assessing the radiographs and medical records at the final outpatient visit. Radiographic OA and severe pain or inability to perform activities of daily living were considered poor outcomes.

Medical complications such as pneumonia, urinary tract infection, and pulmonary embolism and surgical complications such as postoperative foot drop that resulted from sciatic nerve injury, hardware breakage, and reoperation for any reason were assessed.

Radiographic OA, HHS, THA conversion, good/poor outcomes, and complications were compared between the good and poor reduction groups. A subgroup analysis was performed in patients with dome impaction according to the postoperative reduction state, and in patients in the good reduction group according to presence of dome impaction. A multivariate analysis was performed to assess which factors were associated with good/poor outcomes.

### Statistical analysis

Statistical analyses were performed using the SPSS Statistics version 23.0 (IBM Corp., Armonk, NY, USA). We used the chi-square test or Fisher’s exact test for categorical variables, and the independent *t*-test or the Mann–Whitney test for continuous variables. A logistic regression analysis was conducted for the multivariate analysis. First, a bivariate analysis was performed for the variables which could be clinically significant. Then, the variables with *P* < 0.2 in the bivariate analysis were included in the multivariate analysis. All continuous data are expressed as means and standard deviations. Significance was set at *P* < 0.05, and Bonferroni correction was applied for multiple comparisons.

## Results

Among 54 patients, 45 patients were in the good reduction group, whereas 9 patients were in the poor reduction group. No significant differences in age (70.7 ± 5.6 vs. 67.1 ± 5.8, *P* = 0.089); male sex (67 and 73%, *P* = 0.696); and other demographic data including BMI, CCI, injury mechanism, T-score and preoperative OA were observed between the two groups (Table 1). The mean follow-up length was 45.0 ± 63 (12–197) in the poor reduction group and 44.0 ± 37.0 (12–151) in the good reduction group. Regarding fracture pattern, the poor reduction group had column (78%), transverse (11%), and T-type (11%) fractures. No significant differences in the fracture patterns, approaches, and negative predictive factors were noted between the two groups (Table 2).

**Table 1:** Baseline demographics.

	Poor reduction group (n = 9)	Good reduction group (n = 45)	P value
Age (years)	70.7 ± 5.6	67.1 ± 5.8	0.089
Sex			0.696
Male	6 (67%)	33 (73%)	
Female	3 (33%)	12 (27%)	
BMI (kg/m^2^)	25.6 ± 2.9	23.9 ± 2.5	0.067
CCI	3.9 ± 1.5	3.9 ± 1.8	0.972
Injury mechanism			1.000
Low energy	2 (22%)	10 (22%)	
High energy	7 (78%)	35 (78%)	
T-score	− 2.5 ± 0.9	− 1.7 ± 1.2	0.110
Preoperative OA (K–L grade)			0.264
0	2 (22%)	5 (11%)	
1	7 (78%)	34 (76%)	
2	0 (0%)	6 (13%)	
Follow-up length (months)	45.0 ± 63.0 (12–197)	44.0 ± 37.0 (12–151)	0.948

*BMI* body mass index, *CCI* Charlson comorbidity index, *OA* osteoarthritis, *K–L grade* Kellgren–Lawrence grade.

**Table 2:** Details on fracture patterns, approaches, and negative predictive factors.

	Poor reduction group (n = 9)	Good reduction group (n = 45)	P value
Fracture patterns			0.177
Anterior column		13 (29%)	
Both column	7 (78%)	18 (40%)	
ACPHT		5 (11%)	
Transverse	1 (11%)	2 (4%)	
Transverse + PW		2 (4%)	
T-type	1 (11%)	5 (11%)	
Approaches			0.234
Ilioinguinal	2 (22%)	17 (38%)	
Kocher–Langenbeck	0 (0%)	5 (11%)	
Percutaneous	0 (0%)	5 (11%)	
Stoppa	7 (78%)	18 (40%)	
Negative predictive factors			
PW involvement	4 (44%)	17 (38%)	0.723
Dome impaction	5 (56%)	15 (33%)	0.266
Femoral head impaction	2 (22%)	2 (4%)	0.125
Posterior dislocation	0 (0%)	1 (2%)	1.000
Central dislocation	1 (11%)	16 (36%)	0.244
QLP separation	3 (33%)	15 (33%)	1.000

*ACPHT* anterior column and posterior hemitransverse, *PW* posterior wall, *QLP* quadrilateral plate.

The good reduction group demonstrated better clinical outcomes (HHS; 82.1 ± 12.4 vs. 68.6 ± 20.5, *P* = 0.022) and lesser proportion of radiographic OA than the poor reduction group (18 vs. 77%, *P* = 0.001). However, no significant difference in THA conversion was observed between the two groups (9 vs. 33%, *P* = 0.081). The good reduction group demonstrated higher proportion of good outcomes than the poor reduction group (89 vs. 22%, *P* < 0.001). No difference in complications was observed between both groups (Table 3).

**Table 3:** Outcome analysis according to reduction state.

	Poor reduction group (n = 9)	Good reduction group (n = 45)	*P* value
Postoperative OA			0.001
No or minimal OA (K–L grades 0, 1, 2)	2 (22%)	37 (82%)	
Radiographic OA (K–L grades 3, 4)	7 (77%)	8 (18%)	
HHS	68.6 ± 20.5	82.1 ± 12.4	0.022
THA conversion	3 (33%)	4 (9%)	0.081
Outcomes			< 0.001
Good	2 (22%)	40 (89%)	
Poor	7 (78%)	5 (11%)	
Complications			
Pneumonia	1 (11%)	2 (4%)	0.428
Urinary tract infection	1 (11%)	3 (7%)	0.529
Pulmonary embolism	1 (11%)	0 (0%)	0.167
Infection	1 (11%)	1 (2%)	0.308
Foot drop	0 (0%)	0 (0%)	
Hardware breakage	0 (0%)	1 (2%)	1.000
Reoperation	0 (0%)	0 (0%)	
Hospital stay (days)	51.6 ± 39.3	25.0 ± 23.8	0.009

*K–L grade* Kellgren–Lawrence grade, *HHS* Harris hip score, *THA* total hip arthroplasty.

**Figure 5:**
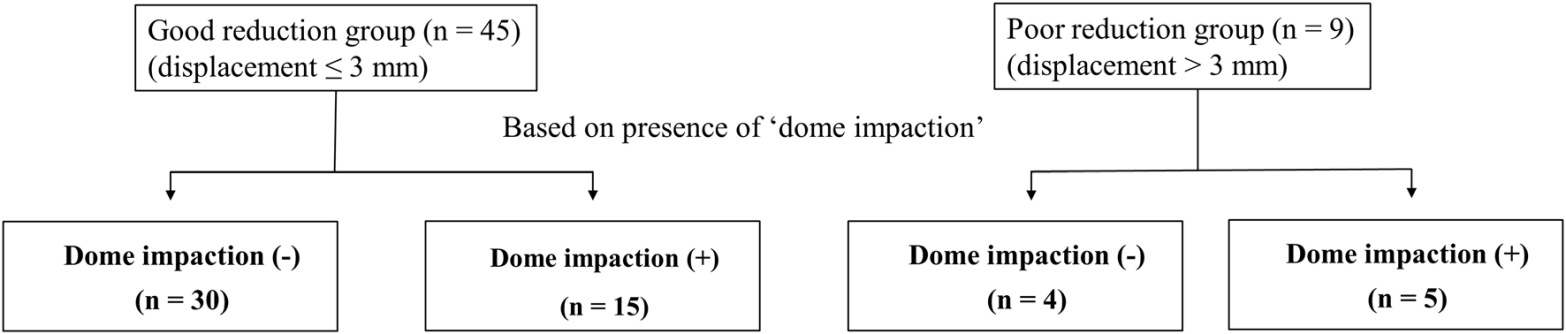
Flowchart of the subgroup analysis in patients with dome impaction according to postoperative reduction state and in the patients in the good reduction group according to presence of dome impaction.

In a subgroup analysis of patients with dome impaction (Fig. 5), radiographic OA, HHS, and THA conversion did not indicate any significant differences between the two groups. Meanwhile, the good reduction group had higher proportion of good outcomes than the displacement group (80 vs. 20%, *P* = 0.031). In a subgroup analysis of the good reduction group, the patients with dome impaction demonstrated a higher incidence of radiographic OA than those without dome impaction (40 vs. 7%, *P* = 0.011). However, HHS, THA conversion, and good/poor outcomes did not demonstrate any significant differences according to the presence of dome impaction (Table 4).

**Table 4:** Subgroup analysis of outcomes.

	Comparison in patients with dome impaction		*P* value
	Poor reduction group (n = 5)	Good reduction group (n = 15)	
Postoperative OA			0.303
No or minimal OA (K–L grades 0, 1, 2)	1 (20%)	9 (60%)	
Radiographic OA (K–L grades 3, 4)	4 (80%)	6 (40%)	
HHS	63.8 ± 20.3	79.8 ± 13.8	0.077
THA conversion	2 (40%)	2 (13%)	0.249
Outcomes			0.031
Good	1 (20%)	12 (80%)	
Poor	4 (80%)	3 (20%)	

	Comparison in the good reduction group		*P* value
	Dome impaction (+) (n = 15)	Dome impaction (−) (n = 30)	
Postoperative OA			0.011
No or minimal OA (K–L grades 0, 1, 2)	9 (60%)	28 (93%)	
Radiographic OA (K–L grades 3, 4)	6 (40%)	2 (7%)	
HHS	79.8 ± 13.8	83.1 ± 11.8	0.447
THA conversion	2 (7%)	2 (13%)	0.591
Outcomes			0.315
Good	12 (80%)	28 (93%)	
Poor	3 (20%)	2 (7%)	

*OA* osteoarthritis, *K–L grade* Kellgren–Lawrence grade, *HHS* Harris hip score, *THA* total hip arthroplasty.

In the bivariate analysis, the female sex, PW involvement, dome impaction, and reduction status were included in the multivariate analysis. In the multivariable analysis, the female sex (odds ratio [OR] 19.335; 95% confidence interval [CI] 1.137–328.851; *P* = 0.040) and poor reduction status (OR 0.014; 95%CI 0.001–0.169; *P* = 0.001) were significantly associated with poor outcomes (Table 5).

**Table 5:** Multiple logistic regression analysis for the risk factors of poor outcomes.

Risk for poor outcomes				
	Bivariate analysis		Multivariate analysis	
	Odds ratio (95% CI)	*P* value	Odds ratio (95% CI)	*P* value
Age	1.041 (0.936–1.158)	0.458		
Female sex	5.950 (1.494–23.701)	0.011	19.335 (1.137–328.851)	0.040
BMI	1.094 (0.858–1.394)	0.467		
CCI	1.163 (0.823–1.643)	0.392		
Preoperative OA (K–L grade)	0.706 (0.189–2.633)	0.605		
Injury mechanism	0.818 (0.183–3.668)	0.793		
Fracture pattern	N/A	0.813		
Negative predictive factors				
PW involvement	2.800 (0.752–10.427)	0.125	6.847 (0.537–87.356)	0.139
Dome impaction	3.123 (0.833–11.703)	0.091	5.379 (0.438–66.091)	0.189
Femoral head impaction	45 (N/A)*	0.002†	N/A	0.999
Posterior dislocation	1.107 (N/A)*	1.000†		
Central dislocation	1.115 (0.284–4.376)	0.876		
QLP separation	1.593 (0.425–5.971)	0.489		
Reduction status	0.036 (0.006–0.222)	< 0.001	0.014 (0.001–0.169)	0.001

*Odds ratio was calculated by adding 0.5 on each cell because the contingency table has one cell containing a zero count.

^†^*P* value was calculated using Fisher’s exact test.

*CI* confidence interval, *BMI* body mass index, *CCI* Charlson comorbidity index, *OA* osteoarthritis, *K–L grade* Kellgren–Lawrence grade, *N/A* not applicable, *QLP* quadrilateral plate.

## Discussion

In the present study, patients with elderly acetabular fractures demonstrated satisfactory outcomes when good reduction was achieved. Additionally, reduction status was the strongest predictor of outcome. In previous studies, dome impaction was proposed as a negative predictive factor, which could suggest later THA in elderly acetabular fractures^10^. However, even with dome impaction, good outcomes could be expected when good reduction was achieved, and patients with good reduction demonstrated comparable outcomes regardless of presence of dome impaction except those with radiographic OA.

Age, delay of surgery, reduction quality, and fracture patterns were identified as prognostic factors for acetabular fractures^20^. Even if age is a prognostic factor, surgical treatment showed better clinical outcomes than non-operative treatment for displaced acetabular fractures in elderly patients^25^. In contrast to other patient predisposing factors, reduction quality could be controlled by the surgeon’s efforts. Thus, studies have emphasized the importance of the reduction of acetabular fractures^10,20–22^. Here, the good reduction group demonstrated satisfactory radiographic and functional outcomes in the overall analysis compared to the poor reduction group. Although acute or delayed THA could be an alternative treatment for elderly acetabular fractures, ORIF can be the first option when appropriate reduction is feasible.

We demonstrated that the conversion rate to THA in patients with dome impaction was 20% (4/20), which was comparative with those of previous studies reporting a conversion rate of approximately 14%–33%^13,23,24^. In contrast to outcomes according to reduction in dome impaction, the poor reduction group (80%) had a higher incidence of poor outcomes than the good reduction group (20%). Dome impaction is a representative prognostic factor, and frustrating outcomes occurred in those who did not use the intrapelvic approach, in the initial report^11^. Consequently, surgical techniques which directly visualize and reduce dome fragments through the intrapelvic approach were introduced^13,25^. Likewise, to address dome impaction, we used mainly the Stoppa (46%) and ilioinguinal (35%) procedures during which we used a third window to allow examination of the intrapelvic structure. Garner and Sagi have reported a reduction of dome impaction using the intrapelvic approach, however they created a cortical window to achieve direct reduction of impacted dome fragments^26^. By contrast, we used a fracture window to expose the dome fragment. In elderly patients, maintenance of reduction in osteoporotic bones was another concern. We supported articular fragments with a rafting screw, which does not rely on osteoporotic cancellous bone but on articular fragments which had a stiff portion. Therefore, bone grafting in defects created by reducing impacted fragments, which could be emphasized in elderly patients, has become a concern. Because no direct comparative study was conducted, filling the defect depends on the surgeon’s preference. Zhuang et al. have reported satisfactory outcomes in patients with dome impaction with iliac bone grafts in bone defects^24^. By contrast, Casstevens has reported the maintenance of reduction of dome impaction using a rafting screw without bone grafts in void^12^, and we also did not perform defect filling using a bone graft or bone substitute.

The benefit of acute THA has been reported as no further surgical procedure was needed after THA. Borg et al. have reported a survival rate of 100% in 13 patients with acute THA^27^. However, if anterior approach is used for stabilizing acetabular fractures, the patient needs to be repositioned and redraped. Herscovici et al. investigated elderly patients with acetabular fractures who underwent concomitant ORIF and THA, and patients who used the ilioinguinal approach for acetabular fracture required an average surgical time of 427 min and an average of 2225 mL compared to those with the Kocher–Langenbeck approach, who required 121 min and 777 mL^28^. Although having a long joint survival with a single operation is important, we focused more on the stabilization of a patient’s condition after major trauma. In the present study, we did not consider the posterior wall and/or column fractures which should be treated through the posterior approach because such fracture types inherently cannot obtain superomedial dome impaction. Comparison with acute THA would be appropriate with the cases mainly operated through the posterior approach.

In the multivariate analysis, the female sex and poor reduction status were associated with poor outcomes. The importance of reduction status has already been previously emphasized^11,13,25^. The female sex has been identified by Lundin et al. as a risk factor of reoperation for acetabular fractures^29^. This result could be explained by bone quality related to postmenopausal osteoporosis. Another hypothesis is the differences in pelvic shape according to sex^30^. Femoral head impaction cannot be analyzed in the multivariate analysis due to zero incidence of patients with both good outcomes and femoral head impaction, and future studies with a large number of patients are needed.

The present study has several limitations. First, the retrospective study had a small sample size: the poor reduction group had a smaller number of patients than the good reduction group, which can create a statistical weakness^31^. For example, the THA conversion did not obtain a statistically significant difference (*P* = 0.081), but an obvious difference can be observed in the value (33% in the poor reduction group vs. 9% in the good reduction group). This is also true for postoperative OA (80 vs. 40%, *P* = 0.303) and HHS (63.8 vs. 79.8, *P* = 0.077) in patients with dome impaction and PW involvement (OR, 6.847; *P* = 0.139) or dome impaction alone (OR, 5.379; *P* = 0.189) in the multivariate analysis. In clinical practice, a poorly reduced articular fracture can lead to radiological OA and poor function, which can result in THA conversion. Thus, we believe that the results were underpowered in this study and should not be overlooked. A future study with a large sample size is required to confirm our results.

Second, although we assessed the reduction status of the weight-bearing dome, cases without dome impaction were evaluated using another area rather than the weight-bearing dome. Because we included the cases without dome impaction, this point was inevitable. Third, dome impaction was analyzed dichotomously, not quantitatively. However, the size and initial displacement of dome impaction were all different. Nevertheless, the present study is valuable in that it is the first study to directly compare the reduction of dome impaction in elderly acetabular fractures.

In conclusion, elderly acetabular fractures demonstrated favorable outcomes when adequate reduction was achieved even with dome impaction. Fracture reduction should be addressed in elderly acetabular fractures with dome impaction.

## Data Availability

The data presented in this study are available on request from the corresponding author. The data are not publicly available due to conditions of the ethics committee of our university.
